# Ecological Partitioning and Body Size Differentiation Enable Coexistence of Closely Related Semi‐Arboreal *Therates* Tiger Beetles (Coleoptera: Cicindelidae)

**DOI:** 10.1002/ece3.72499

**Published:** 2025-11-17

**Authors:** Dale Ann P. Acal, Anna Sulikowska‐Drozd, Radomir Jaskuła

**Affiliations:** ^1^ Faculty of Biology and Environmental Protection, Department of Invertebrate Zoology & Hydrobiology University of Lodz Lodz Poland

**Keywords:** Adephaga, microhabitat preferences, morphological divergence, niche partitioning, Philippines, sympatric species

## Abstract

Mechanisms facilitating the coexistence of ecologically similar species remain a fundamental question in ecology. Here, we investigate niche partitioning among coexisting *Therates* tiger beetles by examining morphological differentiation, temporal activity, altitude distribution, and foraging substrate preferences. Across 40 sites, we analyzed 1065 specimens from six taxa, measuring body size and mandible length. Among these, 18 sites had coexisting species, while 22 had a single species. In total, four pairs of co‐occurring semi‐arboreal *Therates* species were noted. In 13 of the coexistence sites, we also measured leaf dimensions of primary hunting substrates. Our results reveal significant differences in body size and mandible length among coexisting species, with larger species preferring larger leaves. Notably, significant size differences between coexisting and noncoexisting populations of *T. fulvipennis bidentatus* and *T. coracinus coracinus* support the role of character displacement in resource partitioning. Our findings suggest that morphological divergence, microhabitat preferences and spatiotemporal differentiation contribute to coexistence in the studied tiger beetle species. This study supports the niche partitioning hypothesis as a mechanism for coexistence among ecologically similar species. Understanding these mechanisms is critical for biodiversity conservation and management of riparian tropical ecosystems.

## Graphical abstract

Our findings demonstrate that body size, mandible length, spatiotemporal activity, and foraging substrate preferences contribute to niche partitioning and species coexistence among *Therates* tiger beetles. Notably, significant size differences between coexisting and noncoexisting populations support the role of character displacement in resource partitioning. These results align with niche theory, suggesting that species coexist by differentiating their ecological niches across multiple dimensions.

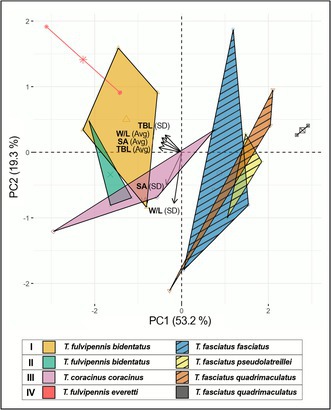

## 1 Introduction

Species coexistence is a fundamental ecological process that maintains biodiversity and ecological stability. It is predicated on the idea that each species occupies a unique ecological niche (Schoener 1974). Habitat heterogeneity and niche differentiation are key drivers of biodiversity, enabling the coexistence of closely related sympatric species (e.g., Estevo et al. 2017; Hardin 1960; Leibold and McPeek 2006; Sanches et al. 2022; Tang and Zhou 2011). A species' niche includes the ecological conditions necessary for its survival, along with its interactions with the environment and other organisms (Chase and Leibold 2004; Hutchinson 1957; Leibold 1995). Hutchinson (1957) defined the niche as the multidimensional space of environmental conditions in which a species can persist. Niche theory suggests that a species' fundamental niche represents the full range of conditions that support its survival and reproduction (Grinnell 1917; Schoener 1974). When species occupy overlapping resource niches, competition arises, often leading to niche differentiation to reduce competition. This aligns with the competitive exclusion principle (Gause 1934), which asserts that no two species can occupy the same niche indefinitely. In the absence of competitors, species may broaden their ecological roles, whereas competition drives increased niche specialization. This process can drive character displacement, where co‐occurring species evolve morphological or behavioral differences to reduce competition (Pfennig and Pfennig 2012).

As active visual predators tiger beetles (Coleoptera: Cicindelidae) are a classic model system for investigating coexistence mechanisms in a broad range of predator communities. Numerous species often occur sympatrically, making them ideal for studying how closely related taxa partition resources (Pearson and Vogler 2001). Numerous studies have shown that predator communities often exhibit morphological differentiation, particularly in traits related to resource acquisition, such as body size and feeding structures (Pearson and Juliano 1991; Pearson and Mury 1979). For instance, mandible size and shape are strongly correlated with prey size and are considered crucial for reducing competition for food among Cicindelidae species that coexist in the same microhabitat during the same time of year and period of the day (Ganeshaiah and Belavadi 1986; Pearson 1980; Pearson and Knisley 1985; Pearson and Mury 1979; Pearson and Stemberger 1980). However, a significant knowledge gap exists. While extensive research has explored coexistence in temperate, ground‐dwelling (epigeic) tiger beetles, our understanding of these mechanisms in tropical regions, especially for semi‐arboreal species remains limited.

This is a critical oversight, as tropical forests harbor a disproportionately high number of species and represent a unique ecological context. In these environments, where open sandy areas are scarce, large tree trunks or lower vegetation with large leaves serve as alternative flat hunting surfaces for arboreal and semi‐arboreal Cicindelidae species (Marohomsalic et al. 2021; Pearson 1980, 1988). The choice of foraging substrate is a crucial aspect of a predator's ecology, influencing hunting success, habitat preferences, and overall fitness (Pearson and Knisley 1985). Known for their active hunting strategies, tiger beetles search for and chase prey (Gilbert 1997; Pearson and Vogler 2001; Rewicz and Jaskuła 2018). Typically, a tiger beetle locates its live prey visually, initiating pursuit through active running, interspersed with pause‐and‐look behavior (Gilbert 1997; Rewicz and Jaskuła 2018). Flat, open surfaces, such as bare soil or sand dunes, provide the best vantage point for small visual predators. These surfaces are preferred by many epigeic Cicindelidae species due to their stable substrates, which enhance hunting effectiveness (Pearson and Vogler 2001).

The genus *Therates* Latreille, 1817, represents a diverse group of semi‐arboreal tiger beetles found in the Oriental region, typically on low vegetation along riparian forest areas (Anichtchenko and Wiesner 2022; Anichtchenko and Medina 2024; Lin and Wiesner 2022; Matalin and Wiesner 2023; Medina et al. 2022; Wiesner 2020). These species are fast, diurnally active forest beetles often observed on the leaves of undergrowth plants and occasionally in habitats such as wooded paths and rocks along forest streams, suggesting their role as understory predators in forest ecosystems (Acal et al. 2021, 2024; Lin and Wiesner 2022). Despite the genus being known for over 200 years, autoecological data, including microhabitat preferences and patterns of species co‐occurrence within communities, remain limited.

This study addresses this gap by quantifying, for the first time, the multidimensional niche differentiation among semi‐arboreal *Therates* tiger beetles in tropical forests. Specifically, we aim to determine whether niche partitioning among coexisting *Therates* species is driven by morphological differentiation and habitat segregation. On foraging substrates such as understory leaves, species may face unique challenges in locating mates and food sources due to the visual complexity of these environments, in contrast to the open habitats typically inhabited by ground‐dwelling tiger beetles. Within these habitats, the beetles' preference for certain leaf sizes as foraging substrates may play a role in reducing resource competition between closely related species. We hypothesize that spatiotemporal activity, morphological divergence, and foraging substrate preferences collectively facilitate coexistence among semi‐arboreal tiger beetles. Our objectives were: (1) to compare the phenological activity and altitudinal distribution of the studied species to determine the extent of their overlap in time and space; (2) to investigate morphological divergence among coexisting species in traits linked to resource acquisition (total body length and mandible size) including a detailed analysis of allometric scaling. We hypothesize that differences in body size between coexisting species contribute to reduced competition through differential prey size preferences (i.e., different mandible sizes correspond to different prey size); (3) to examine the relationship between beetle body size and their preferred leaf size used as a hunting substrate. We hypothesize that coexisting species utilize distinct microniches, in terms of leaf sizes, as a strategy to reduce competition; and (4) to explore potential ecological character displacement by comparing morphological variation among conspecifics from coexisting and noncoexisting populations.

## 2 Methods

### 2.1 Sampling Area

**FIGURE 1:**
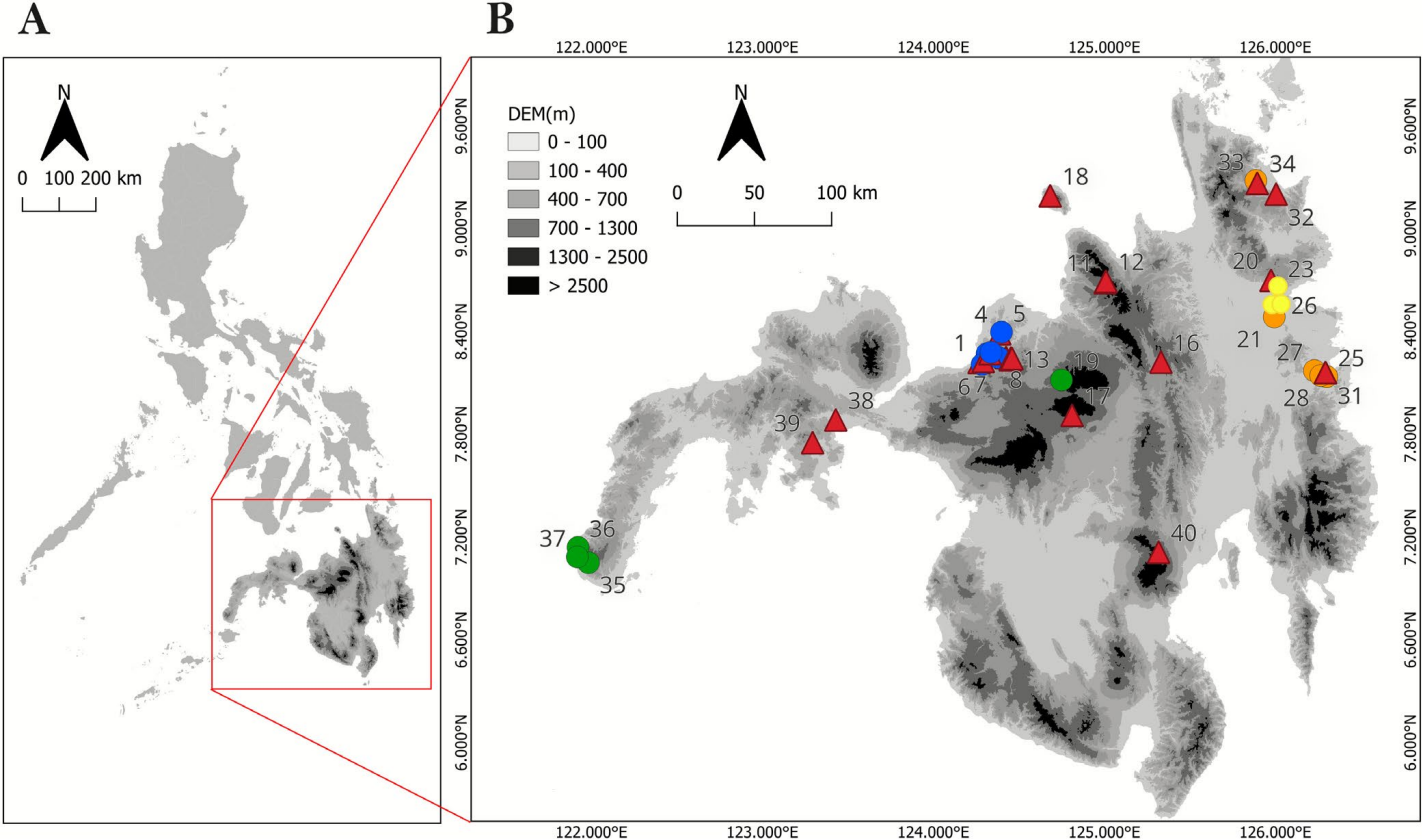
(A) Philippine archipelago. (B) Geographical distribution of sampling sites in southern Philippines (see Table 1 for site details). Circles are sites with species noted in coexistence *T. fulvipennis bidentatus* and *T. fasciatus fasciatus* (blue), *T. fulvipennis bidentatus* and *T. fasciatus pseudolatreillei* (green), *T. coracinus coracinus* and *T. fasciatus quadrimaculatus* (yellow), *T. fulvipennis everetti*, and *T. fasciatus quadrimaculatus* (orange). Red triangles represent those sites where only one species was noted (graphics: D. A. Acal).

The sampling area spans various regions across Mindanao Island in the southern Philippines, including a site on the smaller Camiguin Island (Figure 1, Table 1). In this study, “Mindanao” specifically refers to the mainland, including Camiguin Island, located between 122 to127° E and 5 to 10° N. Mindanao is characterized by a diverse and rugged topography, with prominent mountainous regions, and experiences a tropical climate with distinct wet and dry seasons. Temperatures typically range between 25°C to 32°C. The combination of high temperatures and the proximity to water bodies contributes to a high monthly relative humidity, which varies from 71% to 85% (PAGASA 2023).

**TABLE 1:** Sampling locations of *Therates* taxa in southern Philippines: (A) *T. fulvipennis* *bidentatus*, (B) *T. fasciatus fasciatus* , (C) *T. coracinus coracinus,* (D) *T. fulvipennis* *everetti,* (E) *T. fasciatus* *quadrimaculatus*, and (F) *T. fasciatus* pseudolatreillei.

Sites	Region	Province	Species	GPS coordinates		Elevation (m a.s.l.)
				Latitude	Longitude	
1	Northern Mindanao	Lanao del Norte	A (64 ♂♂, 47 ♀♀)	8.220111	124.2678	72
2	Northern Mindanao	Lanao del Norte	A (6 ♂♂, 5 ♀♀) B (10 ♂♂, 5 ♀♀)	8.271457	124.3141	116
3	Northern Mindanao	Lanao del Norte	A (33 ♂♂, 13 ♀♀)	8.248962	124.4252	352
4	Northern Mindanao	Misamis Oriental	B (15 ♂♂, 10 ♀♀)	8.387611	124.3886	357
5	Northern Mindanao	Misamis Oriental	A (1 ♂, 1 ♀), B (7 ♂♂, 5 ♀♀)	8.396969	124.3984	433
6	Northern Mindanao	Lanao del Norte	A (20 ♂♂, 7♀♀) B (3 ♂♂, 7 ♀♀)	8.207882	124.2848	163
7	Northern Mindanao	Lanao del Norte	A (10 ♂♂)	8.226252	124.2922	118
8	Northern Mindanao	Lanao del Norte	A (16 ♂♂, 6 ♀♀) B (21 ♂♂, 13 ♀♀)	8.249444	124.3792	322
9	Northern Mindanao	Lanao del Norte	A (45 ♂♂, 20 ♀♀)	8.271367	124.3309	119
10	Northern Mindanao	Lanao del Norte	A (26 ♂♂, 9 ♀♀) B (26 ♂♂, 9 ♀♀)	8.279383	124.3374	212
11	Northern Mindanao	Misamis Oriental	C (4 ♂♂, 2 ♀♀)	8.686667	125.005	1170
12	Northern Mindanao	Misamis Oriental	C (15 ♂♂, 4 ♀♀)	8.693333	125.0094	1190
13	Northern Mindanao	Lanao del Norte	F (5 ♂♂, 2 ♀♀)	8.245458	124.4538	914
14	Northern Mindanao	Lanao del Norte	F (6 ♂♂, 2 ♀♀)	8.235767	124.4492	885
15	Northern Mindanao	Lanao del Norte	A (1 ♂, 1 ♀)	8.242511	124.4604	784
16	Northern Mindanao	Bukidnon	F (3 ♂♂, 1 ♀)	8.22175	125.3315	721
17	Northern Mindanao	Bukidnon	B (2 ♂♂)	7.911399	124.8115	1342
18	Northern Mindanao	Camiguin island	F (2 ♂♂)	9.192744	124.6834	693
19	Northern Mindanao	Bukidnon	A (5 ♂♂) F (4 ♂♂, 1 ♀)	7.911484	124.8105	1546
20	CARAGA	Agusan del Sur	E (10 ♂♂, 5 ♀♀)	8.699222	125.9748	170
21	CARAGA	Agusan del Sur	D (6 ♂♂, 5 ♀♀) E (5 ♂♂, 2 ♀♀)	8.485555	125.9937	153
22	CARAGA	Agusan del Sur	C (17 ♂♂, 14 ♀♀) E (18 ♂♂, 14 ♀♀)	8.557953333	125.9807	104
23	CARAGA	Agusan del Sur	C (15 ♂♂, 28 ♀♀) E (9 ♂♂, 3 ♀♀)	8.664923333	126.0157	115
24	CARAGA	Surigao del Sur	C (17 ♂♂, 18 ♀♀) E (3 ♂♂)	8.14263	126.2516	21
25	CARAGA	Surigao del Sur	D (15 ♂♂, 9 ♀♀) E (36 ♂♂, 20 ♀♀)	8.137289	126.301	48
26	CARAGA	Surigao del Sur	C (2 ♂♂, 7 ♀♀) E (1 ♂)	8.5603333	126.0334	108
27	CARAGA	Surigao del Sur	D (2 ♂♂) E (9 ♂♂, 3 ♀♀)	8.172222	126.2286	70
28	CARAGA	Surigao del Sur	D (6 ♂♂, 3 ♀♀) E (6 ♂♂, 3 ♀♀)	8.143386	126.2657	60
29	CARAGA	Surigao del Sur	E (8 ♂♂, 3 ♀♀)	8.16110556	126.2932	53
30	CARAGA	Surigao del Sur	E (2 ♂♂, 1 ♀)	8.16110556	126.2932	625
31	CARAGA	Surigao del Sur	E (5 ♂♂)	8.16110556	126.2932	52
32	CARAGA	Surigao del Sur	E (17 ♂♂, 9 ♀♀)	9.20267778	126.0048	180
33	CARAGA	Surigao del Sur	D (5 ♂♂) E (1 ♂, 1 ♀)	9.28161	125.8862	80
34	CARAGA	Surigao del Sur	E (2 ♂♂)	9.26596111	125.8941	126
35	Zamboanga Peninsula	Zamboanga del Sur	A (3 ♂♂) F (5 ♂♂, 2 ♀♀)	7.051211	121.9828	354
36	Zamboanga Peninsula	Zamboanga del Sur	A (5 ♂♂, 1 ♀) F (5 ♂♂, 2 ♀♀)	7.140453	121.922	93
37	Zamboanga Peninsula	Zamboanga del Sur	A (18 ♂♂, 6 ♀♀) F (25 ♂♂,4 ♀♀)	7.084567	121.918	185
38	Zamboanga Peninsula	Zamboanga del Sur	B (11 ♂♂, 3 ♀♀)	7.884458	123.4292	175
39	Zamboanga Peninsula	Zamboanga del Sur	B (11 ♂♂, 8 ♀♀)	7.751868333	123.2939	130
40	Davao Region	Davao del Sur	C (37♂♂, 32 ♀♀)	7.112286	125.3176	1218

Adult tiger beetles were collected from 40 sampling sites during the period of October to February in the years 2020 to 2023 (Figure 1, Table 1). All sites were located in riparian habitats, except for two sites where samples were collected from leaves along forest trails and near a cave. The selection of these sites was based on existing literature and our prior personal observations of the genus *Therates*, which is typically found in forested riparian environments (Acal et al. 2021, 2024; Medina 2020). The proximity to rivers ensured a consistent moisture supply, resulting in increased humidity and shaded microenvironments that likely correspond to the ecological preferences of the studied genus. All samples were collected using an entomological hand net, immediately preserved in a 96% alcohol solution, and later identified using taxonomic keys (Acal et al. 2021; Wiesner 1988).

### 2.2 Adult Phenological Activity and Altitudinal Distribution

To investigate the phenological activity and elevational distribution of *Therates* species, we used only primary field data collected during targeted surveys from October to February in the years 2020 to 2023. This approach minimized variability associated with weather, temperature, and collector bias often present in museum or literature‐based records. Not all sites were visited every year due to logistical limitations; however, data were aggregated across years to characterize species activity patterns during the surveyed months (October to February). During each site visit, presence–absence data were collected through active searches using hand nets, with approximately 5 person‐hours of survey effort per site. Phenological activity was assessed by recording the presence of each species at each site during a given month. These presence‐only data were structured as site‐month combinations. To visualize seasonal activity patterns, we constructed violin plots where the width of each violin represents the frequency of occurrences for each species during a particular month.

Elevation and geographical coordinates for each site were recorded using a Garmin eTrex 10 GPS. Elevation was classified into three categories: low altitude/lowlands (< 200 m a.s.l.), mid‐range altitude/highlands (200–800 m a.s.l.), and high altitude/mountains (> 800 m a.s.l.) (Encyclopedia Britannica 2025).

### 2.3 Morphometric Measurements

**FIGURE 2:**
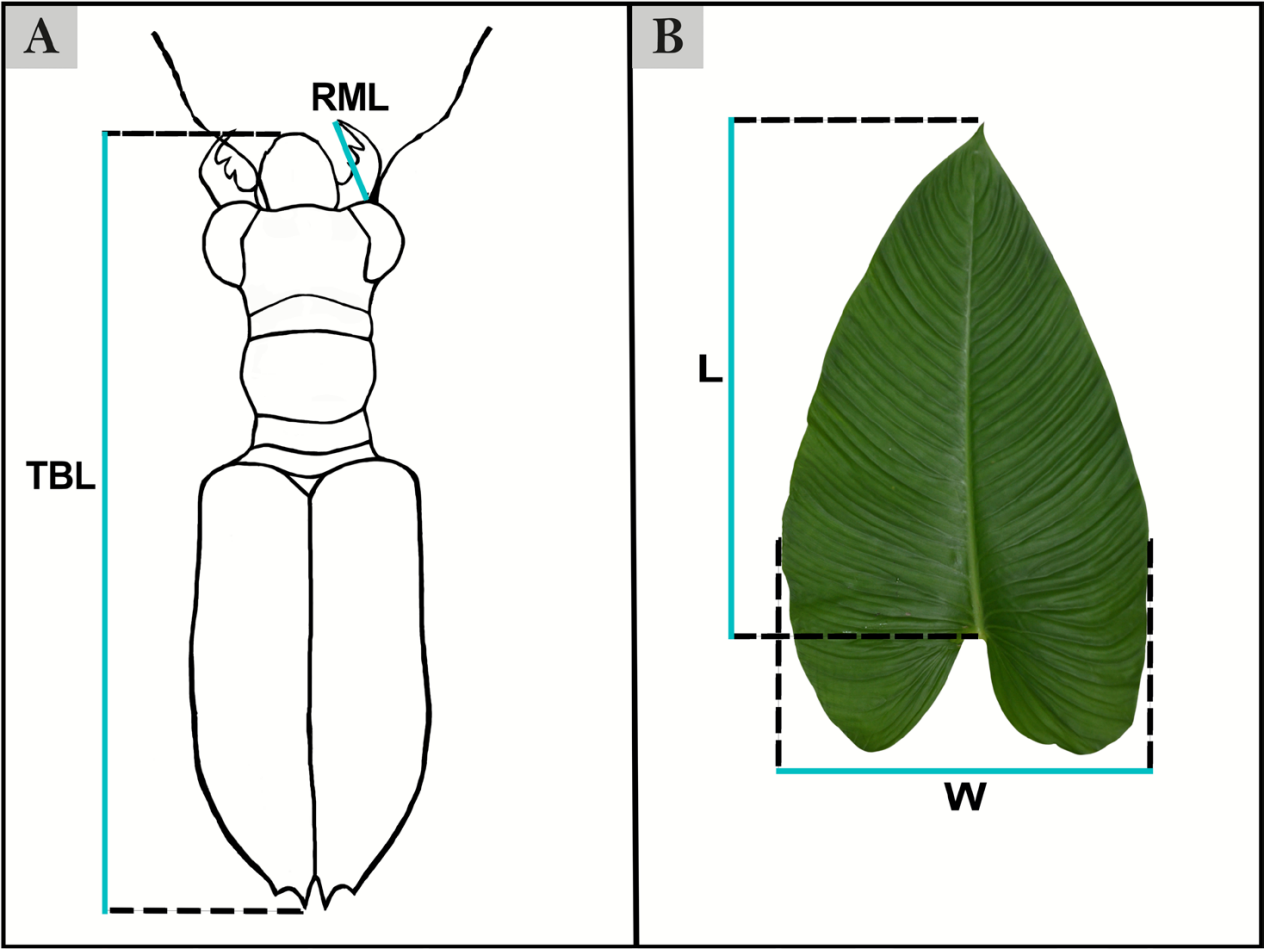
Graphical representation of *Therates* with morphometric parameters (A) Total body length (TBL) and right mandible length (RML). Measured leaf parameters (B) (graphics: D. A. Acal).

A total of 1065 specimens (635♂♂, 361♀♀) were studied, including *T. fulvipennis bidentatus* Chaudoir, 1861 (253♂♂, 115♀♀), *T. coracinus coracinus* Erichson, 1834 (107♂♂, 105♀♀), *T. fulvipennis everetti* Bates, 1878 (34♂♂, 17♀♀), *T. fasciatus fasciatus* Fabricius, 1801 (109♂♂, 60♀♀), *T. fasciatus pseudolatreillei* Horn, 1928 (55♂♂, 14♀♀), and *T. fasciatus quadrimaculatus* Horn, 1895 (132♂♂, 64♀♀). Quantitative measurements (Jaskuła 2005; Acal et al. 2024) included: right mandible length (RML), head length (LH), head width (WH), pronotum width (WP), pronotum length (LP), elytra length (EL), elytra width (WE), labrum length (LL), and total body length (TBL). However, only TBL and RML were retained for the final analysis presented below, as these traits represent key parameters linked to resource partitioning and coexistence mechanisms among co‐occurring species (Figure 2A). Previous studies show that TBL and RML significantly influence prey selection and competition in tiger beetles (Pearson 1980; Pearson and Mury 1979). For sex identification, examination of the forelegs was typically sufficient (the presence of tarsal adhesive setae on the first three tarsomeres of the prothoracic tarsus in males). In cases where further confirmation was needed, sexual copulatory organs were checked (Pearson 1988; Stork 1980). A Nikon SMZ800 stereoscope with a micrometric ocular measurement (Delta optical DLTA12000CM0SSEU3) was used to measure morphological features in millimeters.

### 2.4 Leaf Parameters

In our investigation across 40 sites, 18 were found to have the coexistence of two *Therates* species, while 22 sites had a single species (Figure 1). Among the sites with coexistence, leaves were collected at 13 sampling locations. For each site, 30 leaves were collected per tiger beetle species whenever an active *Therates* individual was observed on the particular leaf surface. The collection and observation period was 12 man‐hours per site. Collected leaves were photographed with a reference scale on a white cloth, and leaf parameters were quantified using ImageJ software version 1.49 (Schneider et al. 2012). High‐resolution leaf images were imported into the software, and calibration was performed using the reference scale present in the images. Regions of interest (ROIs) were defined by outlining the leaf boundaries, and surface area (SA), length (L), and width (W) were measured (Figure 2B). Calibration and measurement procedures were consistently applied across all leaves in the dataset.

### 2.5 Data Analyses

In this study, four coexisting pairs of *Therates* were observed, comprising a total of 597 individuals across the following pairs: (I) *T. fulvipennis bidentatus* (71♂♂, 28♀♀) and *T. fasciatus fasciatus* (70♂♂, 39♀♀), (II) *T. fulvipennis bidentatus* (31♂♂, 7♀♀) and *T. fasciatus pseudolatreillei* (39♂♂, 9♀♀), (III) *T. coracinus coracinus* (51♂♂, 67♀♀) and *T. fasciatus quadrimaculatus* (31♂♂, 17♀♀), and (IV) *T. fulvipennis everetti* (34♂♂, 17♀♀) and *T. fasciatus quadrimaculatus* (57♂♂, 29♀♀).

To assess baseline morphological differences between coexisting species, we used the nonparametric Mann–Whitney U test for total body length (TBL) and right mandible length (RML), supported by boxplots with confidence intervals generated in *ggplot2* (Wickham 2016) and *dplyr* (Wickham et al. 2020). The degree of morphological divergence in mandible length was quantified by calculating the ratio of the mean RML of the larger species to that of the smaller species for each coexisting pair.

To analyze allometric relationships, both RML and TBL measurements were base‐10 logarithmically transformed (log_10_RML and log_10_TBL). This transformation linearizes allometric relationships, allowing for the estimation of allometric exponents (*β*) as slopes in a linear model. Coexisting species pair (SYM) and sex were treated as categorical factors. To assess the combined and interactive effects of total body length, coexisting species pair, and sex on mandible length, a single permutation‐based ANCOVA model was constructed using the *lmp()* function from the *lmPerm* package (version 2.1.4. Wheeler and Torchiano 2025). The model formula was log_10_RML∼log_10_TBL × SYM × Sex, which includes all main effects and all two‐way and three‐way interaction terms. Permutation tests were employed to calculate *p*‐values, which are robust to violations of normality and homogeneity of variances (as determined by Shapiro–Wilk and Levene's tests on raw data). A total of 20,000 permutations were conducted, and a random seed (set. seed(123)) was set to ensure reproducibility of the permutation results. Overall significance for each term in the ANCOVA was obtained by applying the *anova()* function to the lmp model object.

Given the presence of significant interaction terms in the combined ANCOVA, particularly the log_10_TBL × SYM and the borderline significant log_10_TBL × SYM × Sex interactions, separate permutation‐based linear regressions were performed for each unique combination of coexisting species pair and sex (8 groups in total). For each of these group‐specific models, the allometric exponent (βlog TBL), residual standard error, adjusted *R* ^2^, and permutation‐based *p* value were extracted to provide detailed insights into allometric patterns within specific contexts. To assess the general scaling relationship across all studied taxa, we performed linear regression analyses, fitted separately for males and females. For each regression, we tested the significance of the slope and reported the coefficient of determination (*R* ^2^) to quantify the proportion of variance in RML explained by TBL. Additionally, body parameters were standardized by dividing the measured values by the total body length of each individual. To assess leaf morphological variability related to each coexisting species, leaf parameters, including surface area and width‐to‐length aspect ratio, were used to perform a Principal Component Analysis (PCA). The statistical significance of leaf variations chosen by coexisting species was assessed using a one‐way PERMANOVA with the *adonis* function from the *vegan* package. To investigate the potential relationship between beetle body size and preferred leaf sizes for hunting, PCA was employed. This analysis utilized the average and standard deviation of the beetle's total body length, along with leaf parameters, including surface area and the width‐to‐length aspect ratio, at each site where coexistence was observed. The goal of this analysis was to identify patterns in leaf parameters that may contribute to coexistence and niche partitioning among beetle species. Lastly, boxplots were constructed to compare the variation in body sizes among conspecifics collected with co‐occurring species and those collected from sites without coexistence. This was done for all studied species, except for *T. fulvipenns everetti* due to the lack of data for this species in noncoexisting sites. Nonparametric Mann–Whitney U tests were used to assess significant differences within populations of the same sex. All statistical analyses were performed using RStudio software (v. 5.2.4).

## 3 Results

### 3.1 Phenology and Elevational Preference

**FIGURE 3:**
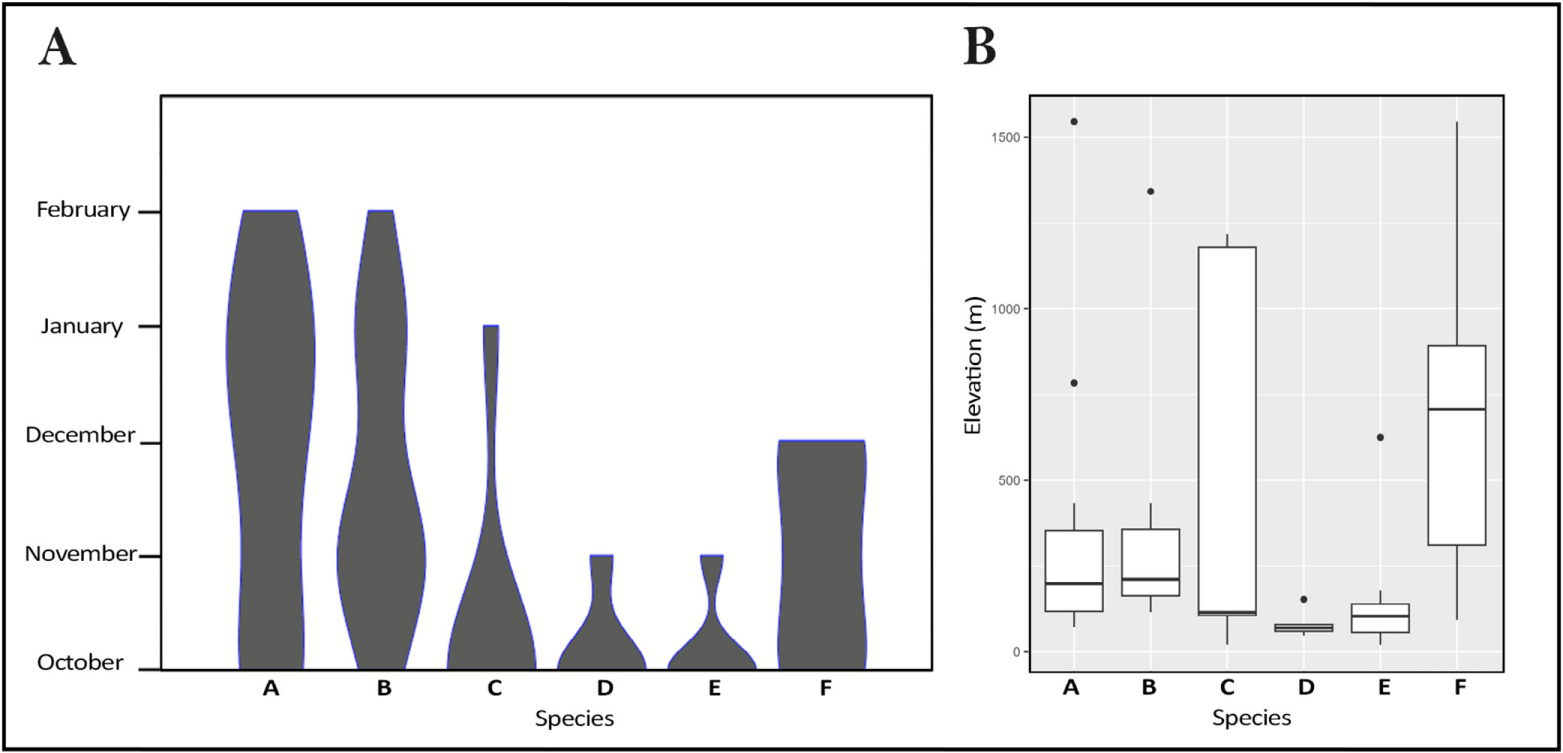
Phenology (A) and elevational distribution (B) of each studied *Therates* taxa: (A) *T. fulvipennis* *bidentatus*, (B) *T. fasciatus fasciatus* , (C) *T. coracinus coracinus*, (D) *T. fulvipennis* *everetti*, (E) *T. fasciatus* *quadrimaculatus*, and (F) *T. fasciatus* pseudolatreillei. Violin plot widths represent the species occurrences across sites in each month.

The six *Therates* species displayed phenologically distinct adult activity patterns across the surveyed period (October to February). *T. fulvipennis bidentatus* and *T. fasciatus fasciatus* showed prolonged activity, recorded consistently throughout all the sampled months. *T. coracinus coracinus* was most frequently encountered from October to November, while *T. fulvipennis everetti* and *T. fasciatus quadrimaculatus* were primarily observed only in October. Lastly, *T. fasciatus pseudolatreillei* exhibited continuous activity from October to December (Figure 3A).

Additionally, among the studied taxa varying altitude preferences were observed across the surveyed gradient. Mid‐range altitudes (200 to 800 m a.s.l.) were preferred by most of the studied taxa, including *T. coracinus coracinus*, *T. fasciatus fasciatus* , *T. f. pseudolatreillei*, and *T. fulvipennis bidentatus*. However, only *T. coracinus coracinus* and *T. fasciatus pseudolatreillei* were observed at high altitudes (> 800 m a.s.l.). In contrast, *T. fulvipennis everetti* and *T. fasciatus quadrimaculatus* showed a preference for lower altitudes (< 200 m a.s.l.), indicating a preference for lowland habitats. The most opportunistic species was *T. coracinus coracinus* which was distributed across the entire elevational gradient (Figure 3B).

### 3.2 Morphological Differences Among Coexisting Species

**FIGURE 4:**
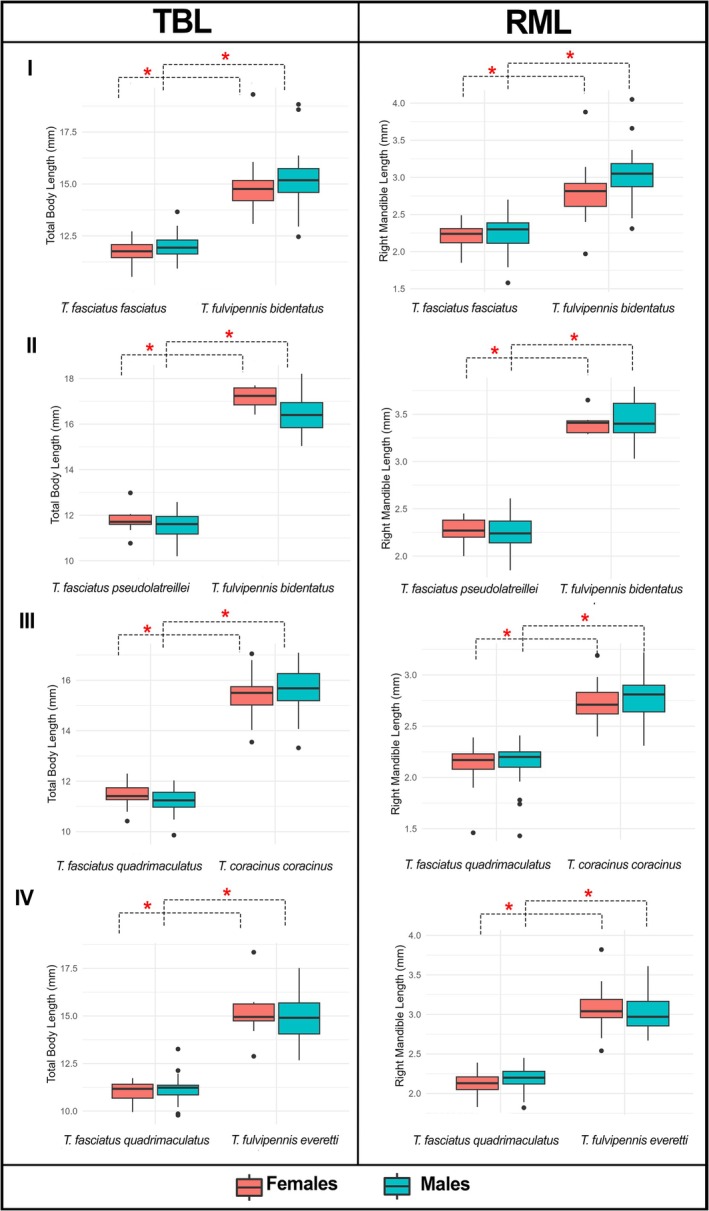
Total body length (TBL) and right mandible length (RML) of males and females of the studied coexisting *Therates*: (I) *T. fulvipennis bidentatus* and *T. fasciatus fasciatus* , (II) *T. fulvipennis bidentatus* and *T. fasciatus* *pseudolatreillei*, (III) *T. fasciatus quadrimaculatus* and *T. coracinus coracinus*, (IV) *T. fasciatus quadrimaculatus, and T. fulvipennis everetti*. The boxes represent the interquartile range (IQR), with the median shown as the horizontal line within the box: Red—females, blue—males. Mann–Whitney U tests: (*p < 0.001).

Significant size differences in total body length (TBL) and right mandible length (RML) were observed between coexisting *Therates* species pairs, consistent across both sexes. In all four coexisting pairs, one species was significantly larger in body size and mandible length than the other, across both sexes (Figure 4). *T. fulvipennis bidentatus* was larger than both *T. fasciatus fasciatus* and *T. fasciatus pseudolatreillei*. Similarly, *T. coracinus coracinus* and *T. fulvipennis everetti* were larger than *T. fasciatus quadrimaculatus* (detailed Mann–Whitney U test results are provided in Supporting Information).

The calculated mandible length ratios for the coexisting species pairs ranged from 1.2808 to 1.5269. Coexisting pair III (*T. coracinus coracinus* and *T. fasciatus quadrimaculatus*) had a ratio of 1.2808, while the remaining three pairs (I, II, IV) consistently exceeded the 1.3 threshold typically observed in coexisting epigeic tiger beetles, with ratios ranging from 1.3279 to 1.5269 (Table 2).

**TABLE 2:** Mandible length ratios for each coexisting *Therates* species pairs.

Coexisting pair	Larger species (RML mean)	Smaller species (RML mean)	Interspecific mandible ratio
I	2.970505	2.236972	1.327913
II	3.438421	2.251875	1.526915
III	2.741356	2.140417	1.280758
IV	3.066078	2.169767	1.413091

*Note:* (I) *T. fulvipennis bidentatus* and *T. fasciatus fasciatus* , (II) *T. fulvipennis bidentatus* and *T. fasciatus* *pseudolatreillei*, (III) *T. coracinus coracinus* and *T. fasciatus quadrimaculatus*, (IV) *T. fulvipennis everetti* and *T. fasciatus quadrimaculatus*.

The allometric scaling of mandible length was analyzed using permutation‐based ANCOVA. The model revealed highly significant main effects of total body length (log_10_TBL) (*F* = 3335.41, *p* < 2.2e‐16), indicating that mandible length strongly and predictably scales with overall body size. Beyond this fundamental scaling, both coexisting pairs SYM (*F* = 51.24, *p* < 2.2e‐16) and sex (*F* = 10.58, *p* = 0.001) significantly influenced mandible length, suggesting baseline differences related to species co‐occurrence and sexual dimorphism, respectively. Crucially, a highly significant two‐way interaction between log_10_TBL and SYM was detected (*F* = 17.22, *p* = 9.98e‐11). This indicates that the allometric slope of mandible length varies significantly depending on the specific coexisting species pair, implying context‐dependent allometric patterns. In contrast, neither the log_10_TBL × Sex (*F* = 2.15, *p* = 0.14) nor the SYM × Sex (*F* = 0.43, *p* = 0.73) interactions were statistically significant, suggesting that the allometric slope does not generally differ between sexes, and baseline sexual dimorphism is relatively consistent across coexisting pairs. A three‐way interaction, log_10_TBL × SYM × Sex, was observed to be borderline significant (*F* = 2.49, *p* = 0.059), hinting at a more complex pattern where the variation in allometric slopes among coexisting species pairs might be subtly modulated by sex (Table 3).

**TABLE 3:** Analysis of variance on permutation‐based ANCOVA, examining the effects of total body length (log_10_TBL), coexisting species pair (SYM), and sex on right mandible length (log_10_RML) in the studied *Therates*.

Source of variation	df	*R* Sum Sq	*R* Mean Sq	*F*	Pr(>*F*)
log_10_TBL	1	2.26863	2.26863	3335.4147	< 2.2e‐16***
SYM	3	0.10456	0.03485	51.2429	< 2.2e‐16***
log_10_TBL:SYM	3	0.03515	0.01172	17.2245	9.98E‐11***
Sex	1	0.0072	0.0072	10.5808	0.001209**
log_10_TBL:Sex	1	0.00146	0.00146	2.1507	0.143045
SYM:Sex	3	0.00087	0.00029	0.4256	0.734731
log_10_TBL:SYM:Sex	3	0.00509	0.0017	2.4932	0.059166*
Residuals	581	0.39518	0.00068		

*Note:* *F*‐statistics and *p* values (Pr(>*F*)) for each term are derived from permutation tests using the anova() method on the lmp model object with 20,000 permutations. ***p < 0.001; **p < 0.01; *p < 0.1.

To elucidate the significant log_10_TBL × SYM interaction and explore the nuanced three‐way interaction, separate permutation‐based regressions were performed for each of the four coexisting pairs and sex combinations (Table 4). All coexisting pair models were highly significant (*p* < 0.001) and explained a substantial portion of the variance in log_10_RML (Adjusted *R* ^2^ ranging from 0.7684 to 0.9752).

**TABLE 4:** Permutation‐based allometric regressions of right mandible length (log_10_RML) against total body length (log_10_TBL) for each coexisting *Therates* pairs, separated by sex.

Coexisting pair (SYM)	Sex	*N*	Allometric exponent (βlog TBL)	Residual Std. error	Adjusted *R* ^2^	*p* (Pr(Prob))^a^
I	Male	140	1.225	0.03265	0.8181	< 2e‐16***
I	Female	66	1.048	0.02167	0.8754	< 2e‐16***
II	Male	69	1.180	0.02043	0.9556	< 2e‐16***
II	Female	15	1.069	0.01468	0.9752	7.6e‐13***
III	Male	81	0.8258	0.02912	0.8079	< 2e‐16***
III	Female	83	0.8481	0.02577	0.7684	< 2e‐16***
IV	Male	90	1.058	0.02246	0.9097	< 2e‐16***
IV	Female	45	1.111	0.02018	0.9488	< 2e‐16***

*Note:* (I) *T. fulvipennis bidentatus* and *T. fasciatus fasciatus* , (II) *T. fulvipennis bidentatus* and *T. fasciatus* *pseudolatreillei*, (III) *T. coracinus coracinus*, and *T. fasciatus quadrimaculatus*, (IV) *T. fulvipennis everetti* and *T. fasciatus quadrimaculatus*.

^a^ Permutation‐based *p* value for the overall model fit. ***p < 0.001.

The allometric exponents (βlog TBL) varied considerably across these groups, ranging from 0.8258 (SYM III, male) to 1.225 (SYM I, male). This wide range directly confirms that the allometric scaling of mandible length differs significantly across coexisting species pairs (log_10_TBL × SYM). For instance, individuals in coexisting pair III (*T. coracinus coracinus* and *T. fasciatus quadrimaculatus*) (male: βlog TBL = 0.8258; female: βlog TBL = 0.8481) showed approximate isometry or slight negative allometry, indicating that mandible length grew proportionally or even slightly slower than body length. Conversely, individuals in coexisting pair I (*T. fulvipennis bidentatus* and *T. fasciatus fasciatus* ) (male: βlog TBL = 1.225, female: βlog TBL = 1.048), coexisting pair II (*T. fulvipennis bidentatus* and *T. fasciatus pseudolatreillei*) (male: βlog TBL = 1.180; female: βlog TBL = 1.069), and coexisting pair IV (*T. fulvipennis everetti* and *T. fasciatus quadrimaculatus*) (male: βlog TBL = 1.058, female: βlog TBL = 1.111) exhibited varying degrees of positive allometry, with mandibles growing considerably faster than body length.

**FIGURE 5:**
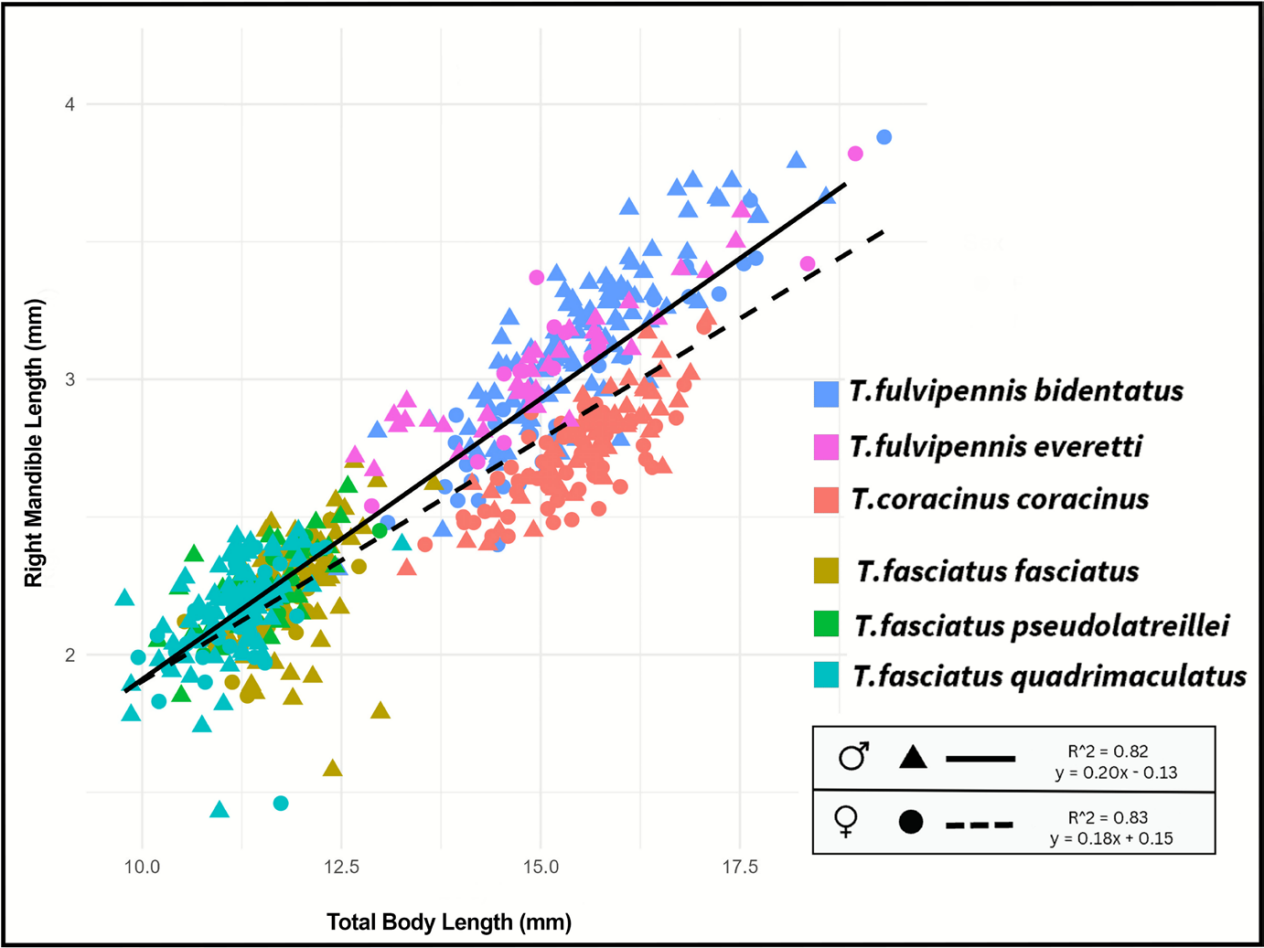
Linear regression analysis of right mandible length and total body length across all studied *Therates* species.

These coexisting pair exponents also provide insights into the borderline three‐way interaction, revealing context‐dependent patterns of sex‐specific allometry. While overall sexual dimorphism in allometric slopes was not significant, the fine‐scale differences within specific coexisting pairs suggest subtle modulations. For example, in coexisting pair I (*T. fulvipennis bidentatus* and *T. fasciatus fasciatus* ) and pair II (*T. fulvipennis bidentatus* and *T. fasciatus pseudolatreillei*), males displayed steeper allometric slopes than females, indicating more pronounced positive allometry in males within these competitive environments. In contrast, in coexisting pair III (*T. coracinus coracinus* and *T. fasciatus quadrimaculatus*), both sexes exhibited similar, more isometric slopes, and in coexisting pair IV (*T. fulvipennis everetti* and *T. fasciatus quadrimaculatus*), females showed a slightly steeper slope than males. Across all studied taxa, a strong positive relationship between TBL and RML was observed in both males (*R* ^2^ = 0.82) and females (*R* ^2^ = 0.83). Interestingly, when examining the overall morphospace occupied by these traits, a gap between around 12.6 and 13 mm of TBL emerged, highlighting a distinctiveness of coexisting species (Figure 5).

**FIGURE 6:**
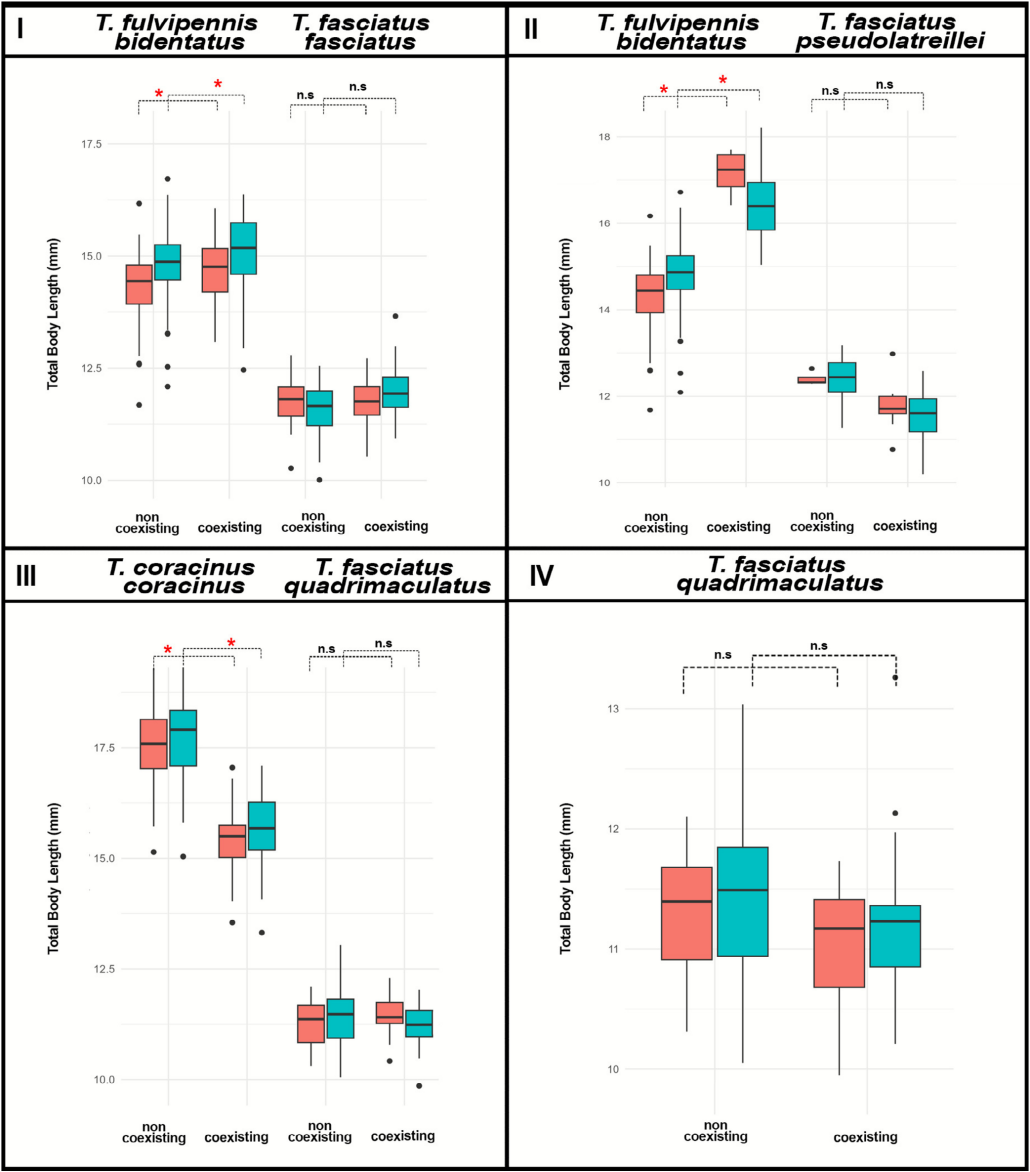
Comparison of total body length in *Therates* found in coexisting and noncoexisting populations. *T. fulvipennis everetti* was excluded due to the lack of data from noncoexisting sites. Red—females, blue—males. Mann–Whitney U tests (*p < 0.001).

Body size comparisons between conspecifics from coexisting and noncoexisting populations revealed significant size differences in two species. In *T. fulvipennis bidentatus*, individuals from coexisting populations were larger than those from noncoexisting populations (pair I: *p* = 2 × 10^−8^, pair II: *p* = 3.36 × 10^−8^). Conversely, for *T. coracinus coracinus*, individuals were larger in noncoexisting populations compared to coexisting populations (pair III: *p* = 2 × 10^−4^). No significant size differences were observed in *T. fasciatus fasciatus* (*p* = 0.366), *T. fasciatus* *pseudolatreillei* (*p* = 0.297), and *T. fasciatus quadrimaculatus* (*p* = 0.855) between coexisting and non‐coexisting populations (Figure 6).

### 3.3 Foraging Substrate Preferences

**FIGURE 7:**
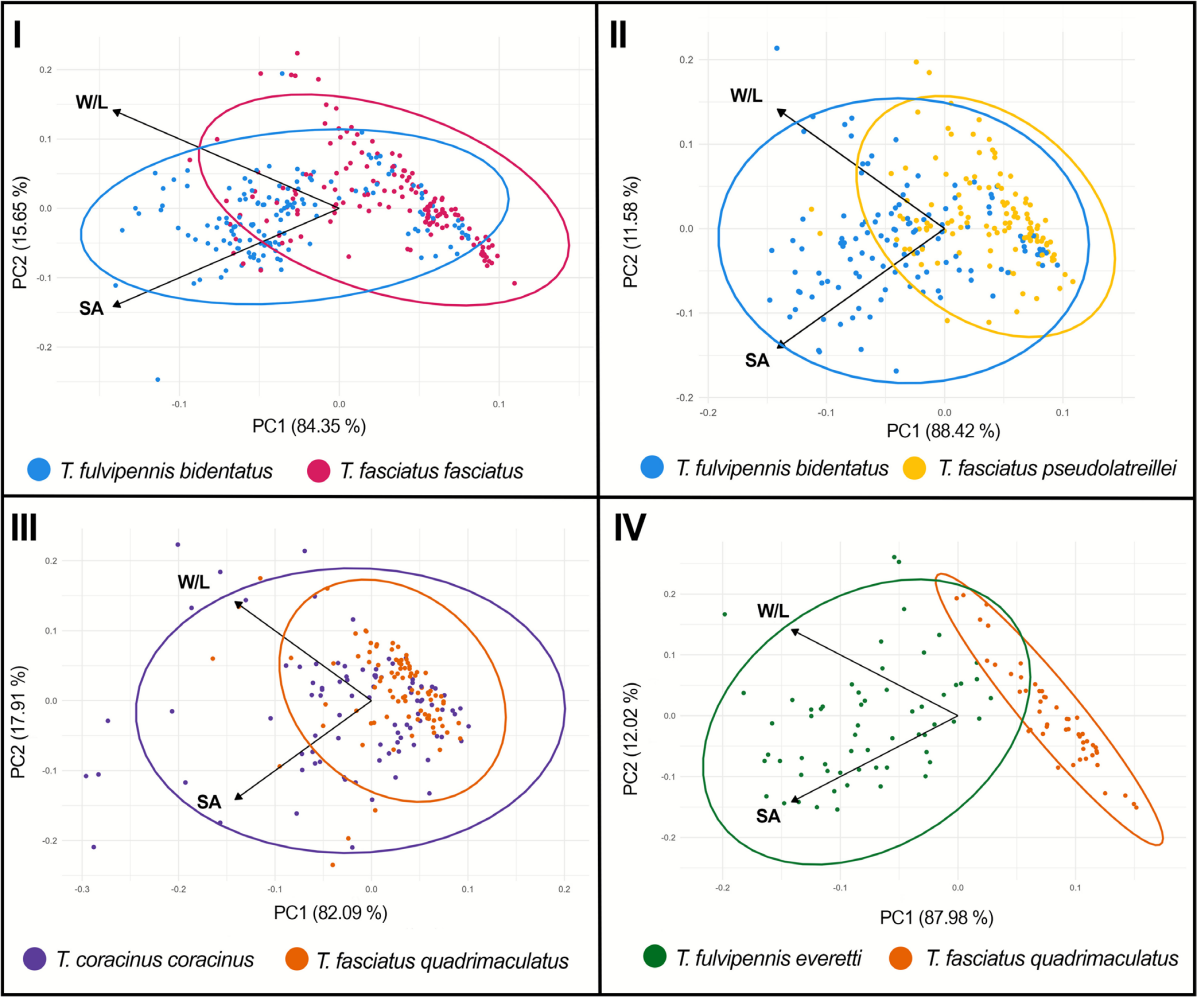
Variations on leaf surface area (SA) and leaf aspect ratio (W/L) between leaves chosen by each coexisting *Therates* species.

**FIGURE 8:**
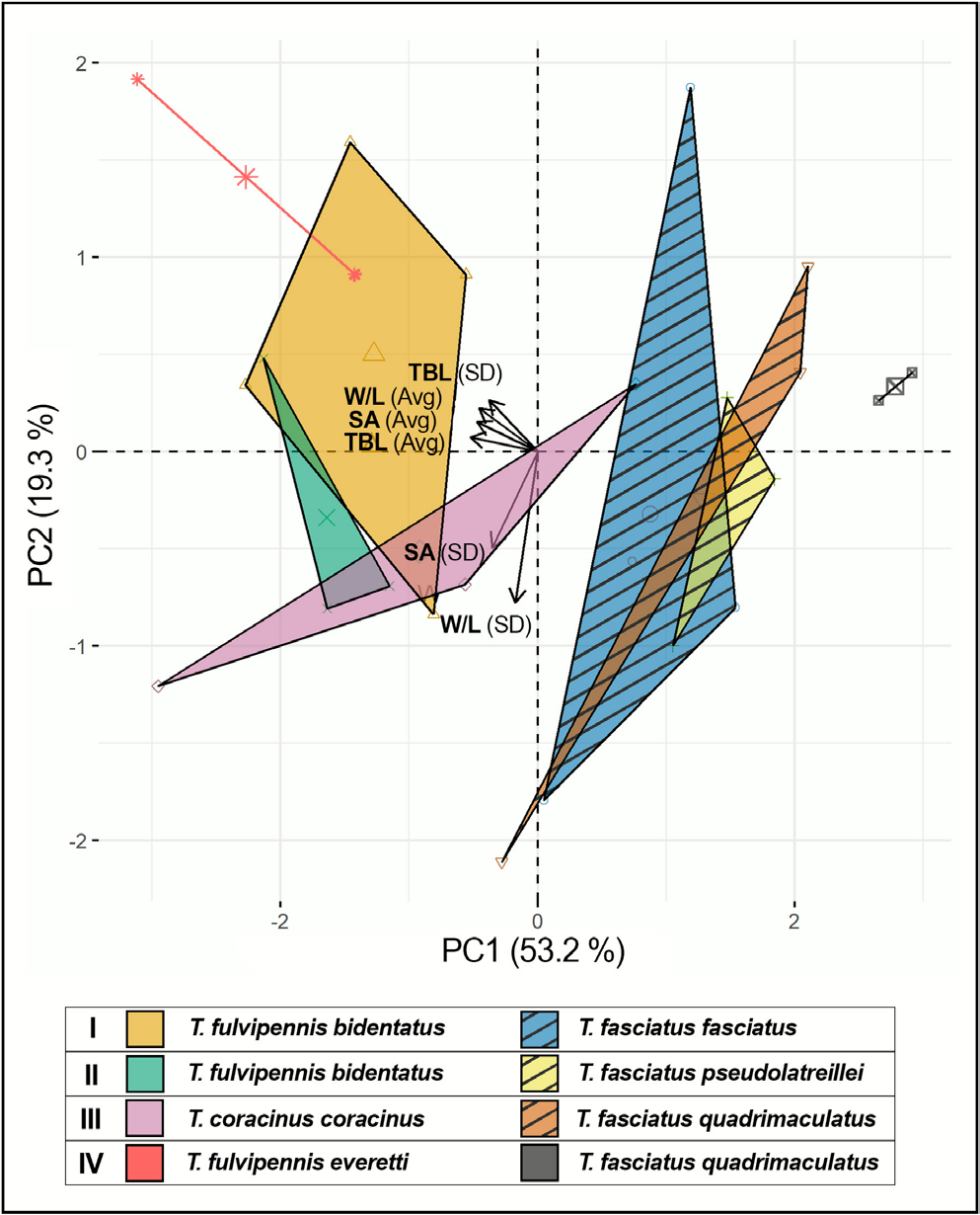
PCA analysis of *Therates* body size and leaf parameters in coexisting species at each site with variables including average (Avg) and standard deviation (SD) of the total body length (TBL), leaf surface area (SA), and leaf aspect ratio (W/L). For IV coexisting pair, *T. fulvipennis everetti* and *T. fasciatus quadrimaculatus* were collected only in two localities.

One‐way PERMANOVA analysis revealed significant differences in leaf parameters among coexisting species pairs. Notable differences were observed between *T. fulvipennis bidentatus* and *T. fasciatus fasciatus* (*F* = 6473.5, *p* < 0.034), *T. fulvipennis bidentatus* and *T. fasciatus pseudolatreillei* (*F* = 8375.2, *p* < 0.006), and *T. fulvipennis everetti* and *T. fasciatus quadrimaculatus* (*F* = 132,339, *p* < 0.001). On the contrary, no significant differences in leaf parameters were observed between *T. coracinus coracinus* and *T. fasciatus quadrimaculatus* (*F* = −34.876, *p* < 0.8) (Table 5). Additionally, PCA results for all coexisting species indicated that the first principal component explained 82% to 88% of the total leaf variation (Figure 7). When examining the relationship between body size and preferred leaf size among coexisting species, PCA revealed that the first two principal components collectively explained 72.5% of the total variation (Figure 8). One‐way PERMANOVA also showed significant differences between coexisting species in both body size and leaf substrate preferences (*F* = 4.17, *p* < 0.0012) (Table 6).

**TABLE 5:** One‐way PERMANOVA results on leaf surface area (SA) and leaf aspect ratio (W/L) between leaves chosen by each coexisting *Therates* species (9999 permutations, Euclidean distance).

Source	df	Sum of Sqs.	*R* ^2^	*F*	*p*
(I) *T. fulvipennis bidentatus* and *T. fasciatus fasciatus*					
Species	1	14,542,906	0.95599	6473.5	0.034
Residual	298	669,469	0.04401		
Total	299	15,212,375	1.00000		
(II) *T. fulvipennis* bidentatus and *T. fasciatus* pseudolatreillei					
Species	1	2,066,464	0.97237	8375.2	0.006
Residual	238	58,723	0.02763		
Total	239	2,125,187	1.00000		
(III) T. coracinus coracinus and *T. fasciatus* quadrimaculatus					
Species	1	−8570	−0.24368	−34.876	0.807
Residual	178	43,741	1.24368		
Total	179	35,171	1.00000		
(IV) *T. fulvipennis* everetti and *T. fasciatus* quadrimaculatus					
Species	1	321,709	0.99911	132,339	0.001
Residual	118	287	0.00089		
Total	119	321,995	1.00000		

**TABLE 6:** One‐way PERMANOVA results on beetle total body length and preferred leaf parameters (leaf surface area [SA] and leaf aspect ratio [W/L]) between coexisting *Therates* species (9999 permutations, Euclidean distance).

Source	df	Sum of Sqs.	*R* ^2^	*F*	*p*
Species	7	64.614	0.64564	4.1645	0.0012
Residual	16	35.464	0.35436		
Total	23	100.079	1		

## 4 Discussion

Competition is a fundamental ecological process that drives evolutionary changes and fosters coexistence, particularly in species‐rich communities like those found in tropical regions where the complexity of biotic interactions increases (Pearson 1980; Schultz and Hadley 1987). Coexisting species typically partition niches across multiple dimensions (spatial, temporal, or trophic) to minimize competitive overlap and facilitate their persistence (e.g., Hutchinson 1957; Hardin 1960; Pianka 1976; Schoener 1974). Our study advances this understanding by providing a comprehensive investigation of niche partitioning among coexisting semi‐arboreal tiger beetle species in the Philippine biodiversity hotspot, with a focus on phenological activity, elevational distribution, morphological divergence (including detailed allometric scaling), and foraging substrate preferences. It provides the first critical data from tropical regions, which have been largely underrepresented in tiger beetle ecological research that has predominantly focused on temperate, ground‐dwelling species (e.g., Carroll and Pearson 1998b, 1998a; Jaskuła 2011, 2015; Jaskuła et al. 2019; Jaskuła and Płóciennik 2020; Knisley and Hill 1992; Pearson and Cassola 1992; Schultz 1989).

Our findings reveal that coexisting *Therates* species exhibit clear patterns of temporal and spatial niche partitioning. While activity among all investigated species overlapped in October and November, only *T. fulvipennis bidentatus* and *T. fasciatus fasciatus* remained consistently active throughout the study period. This suggests that while some temporal co‐occurrence exists, seasonal shifts in activity likely mitigate interspecific competition. Such temporal partitioning is a widely documented adaptation for reducing interspecific competition in tiger beetle communities, including those in tropical areas (e.g., Knisley 1984; Pearson and Derr 1986; Stork and Paarmann 1992; Willis 1967). This strategy, however, remains strongly linked to seasonal cycles in prey abundance (Pearson and Vogler 2001). Regular studies on the phenological activity of adult Cicindelidae in the Philippines are, unfortunately, still lacking. Existing knowledge is primarily based on single records or short‐term, occasional observations restricted to small geographical areas (e.g., Acal et al. 2021; Anichtchenko and Medina 2024; Medina 2020; Medina et al. 2020; Medina et al. 2022). We recognize that the restricted temporal scope of our study limits extrapolation to complete annual phenological patterns and may have resulted in the omission of seasonally active congeners. This limitation highlights the importance of long‐term, year‐round monitoring, especially in the context of ongoing climate change, which is likely to alter insect phenology, range dynamics, and ecological interactions (Schweiger et al. 2008; Vitali et al. 2023).

Spatial partitioning is evident in the differential elevational distribution of *Therates* taxa, consistent with global patterns of altitudinal segregation in tiger beetles, which frequently correspond to adaptation to microclimatic regimes (Pearson and Cassola 1992; Pearson and Vogler 2001). The elevational distribution of the studied *Therates* is characterized by mid‐range preferences, with four taxa occupying between 200 and 800 m a.s.l. Interestingly, the highest frequency of coexisting pairs exhibited a bimodal pattern, occurring at mid‐elevations (pairs: *T. fulvipennis bidentatus* with *T. fasciatus fasciatus*, and *T. fulvipennis bidentatus* with *T. fasciatus pseudolatreillei*) and at lower elevations (< 200 m a.s.l.; pairs: *T. coracinus coracinus* with *T. fasciatus quadrimaculatus*, and *T. fulvipennis everetti* with *T. fasciatus quadrimaculatus*). Notably, *T. coracinus coracinus* was the only eurytopic species, recorded along the entire elevational gradient, a pattern that underscores its broad ecological adaptability and suggests a capacity to tolerate diverse microclimatic conditions and habitat types, in contrast to the more restricted elevational ranges exhibited by its congeners. Surprisingly, this species co‐occurred with only one congener (*T. fasciatus quadrimaculatus*).

Despite the semi‐arboreal niche of *Therates* in forested habitats, their mid‐elevation preference aligns closely with personal data on epigeic Cicindelidae from riparian and sandy habitats on the same island: 11 of 13 species (85%) of these ground‐dwelling tiger beetles also preferred mid‐range altitudes (Acal, Jaskuła—personal data). These findings from the Philippine tropics contrast with the general trend observed in tiger beetle fauna worldwide, where the highest number of species is typically found in lowland areas (e.g., Andriamampianina et al. 2000; Jaskuła 2011, 2015; Pearson and Cassola 1992).

Our results provide strong evidence for morphological niche partitioning, particularly in traits closely associated with resource acquisition. We found significant differences in both body size and right mandible length among all four coexisting species pairs, with one species consistently larger than its counterpart in each pair. This size divergence strongly indicates that body size and mandible length are key traits mediating coexistence in *Therates* species, consistent with patterns observed in other predator communities, where morphological differentiation is linked to prey selection and reduces interspecific competition (Pearson and Mury 1979).

Beyond simple size differences, our detailed allometric analysis revealed context‐dependent allometric scaling of mandibles. The significant interaction between log_10_TBL and coexisting species pair (SYM) indicates that the allometric slope of mandible length varies substantially depending on the specific competitive environment. For instance, species in coexisting pairs I (*T. fulvipennis bidentatus* and *T. fasciatus fasciatus* ), II (*T. fulvipennis bidentatus* and *T. fasciatus pseudolatreillei*), and IV (*T. fulvipennis everetti* and *T. fasciatus quadrimaculatus*) exhibited positive allometry (mandibles growing disproportionately faster than body length), suggesting a strong selective pressure for larger mandibles relative to body size in these competitive contexts. Conversely, coexisting pair III (*T. coracinus coracinus* and *T. fasciatus quadrimaculatus*) showed approximate isometry or slight negative allometry, indicating a different selective strategy. This differential allometric growth offers a mechanistic explanation for niche partitioning, as varying scaling relationships can lead to distinct foraging capabilities and resource exploitation strategies, effectively separating the species' ecological roles even when overall body sizes overlap (e.g., Gould 1966; Schoener 1974; Werner and Gilliam 1984). Further supporting this morphological divergence, the interspecific mandible length ratio (1.28 to 1.53) in three of the four coexisting pairs exceeded the widely recognized 1.3 threshold, a value commonly observed in coexisting epigeic tiger beetles and often cited as a benchmark for character displacement driven by resource competition. This pattern is well‐documented across diverse ecosystems, including desert grassland habitats in the USA (Pearson and Mury 1979), scrub forest floors in India, and tropical forest floors in South and Central America, Borneo, and New Guinea (Pearson 1980). This suggests that morphological divergence in *Therates* mandibles facilitates prey size partitioning, as longer mandibles allow for a wider gape and potentially facilitate the capture of larger prey (Jaskuła 2005; Satoh et al. 2003; Satoh and Hori 2005). However, it is important to note that deviations from this threshold have been observed in habitats with abundant resources and more than three Cicindelidae species, where relaxed competition or alternative mechanisms of resource partitioning may reduce the selective pressure for mandible differentiation (Pearson and Juliano 1991). While body size and mandible length are involved in resource partitioning (e.g., Pearson and Juliano 1991; Pearson and Mury 1979; Pearson and Stemberger 1980; Satoh and Hori 2005; Zerm and Adis 2001), other factors such as shared evolutionary history, prey availability, and habitat disturbance, may also contribute significantly to the observed patterns (Akiyama et al. 2020; Pfennig and Pfennig 2010, 2012).

Our study also elucidates the role of foraging substrate preferences in niche partitioning. The unique semi‐arboreal behavior of *Therates* that predominantly forage on elevated and flat upper leaf surfaces in the shaded undergrowth likely provides a visual advantage over the forest floor. Our study reveals that coexisting *Therates* species exhibit size‐dependent microhabitat specialization: the choice of leaf size as a foraging substrate correlates significantly with the beetles' body size. This pattern suggests an evolutionary alignment of foraging strategy with substrate characteristics. This specialization on different‐sized hunting substrates effectively reduces direct interspecific competition, facilitating the coexistence of multiple species within the same macrohabitat. It simultaneously acts as a strategy to optimize foraging efficiency by allowing species to target substrates likely associated with higher prey densities (Pearson and Vogler 2001).

Finally, we found further strong evidence that morphological differences in co‐occurring *Therates* species facilitate coexistence, as direct comparison of body size between conspecifics from coexisting and non‐coexisting populations revealed significant size shifts consistent with character displacement. In *T. fulvipennis bidentatus*, individuals from coexisting populations were significantly larger than those from noncoexisting populations. This pattern suggests that increased body size serves as a key functional trait for niche differentiation, a widely recognized indicator of character displacement wherein co‐occurring species diverge morphologically to minimize competition (Pfennig and Pfennig 2012; Stuart and Losos 2013). Conversely, in *T. coracinus coracinus*, individuals were significantly smaller in coexisting populations. This contrasting pattern suggests an alternative ecological mechanism at play. In the absence of close congeners, larger body sizes in noncoexisting populations could result from a different competitive or predatory regime, possibly driven by other predatory arthropods. Here, individuals may adopt a growth strategy that optimizes competition for prey or predation avoidance (Pfennig and Pfennig 2012).

In conclusion, our findings demonstrate that spatiotemporal activity, morphological divergence (including allometry and mandible ratios), and foraging substrate preferences, contribute to species coexistence among *Therates* tiger beetles in a tropical hotspot. Notably, significant body size differences between coexisting and noncoexisting populations further support the role of character displacement in facilitating resource partitioning. These results strongly align with and contribute to classic niche theory, illustrating how species coexist by differentiating their ecological niches across multiple dimensions, and providing valuable insights from an understudied tropical, semi‐arboreal system. Further research on adult *Therates* beetles should focus on annual phenology, hunting behavior, and diet using approaches such as molecular gut content analysis and stable isotope techniques Crucially, understanding microhabitat preferences of their largely unknown larvae, and examining their interactions with non*‐Therates* tiger beetles or other predatory arthropods, is essential for a comprehensive understanding of their coexistence dynamics.

## Author Contributions

**Dale Ann P. Acal:** conceptualization (equal), data curation (lead), formal analysis (lead), funding acquisition (equal), investigation (lead), methodology (lead), resources (equal), software (lead), validation (lead), visualization (lead), writing – original draft (lead), writing – review and editing (lead). **Anna Sulikowska‐Drozd:** conceptualization (supporting), funding acquisition (equal), project administration (equal), supervision (equal), validation (equal), visualization (equal), writing – original draft (equal), writing – review and editing (equal). **Radomir Jaskuła:** conceptualization (lead), formal analysis (equal), funding acquisition (equal), investigation (supporting), methodology (lead), supervision (lead), validation (equal), visualization (supporting), writing – original draft (equal), writing – review and editing (equal).

## Conflicts of Interest

The authors declare no conflicts of interest.

## Supporting information


**Data S1:** ece372499‐sup‐0001‐Supinfo.zip.

## Data Availability

The data that supports the findings of this study are available in the Supporting Information of this article.
