# Supplementary material for: Ecological Partitioning and Body Size Differentiation Enable Coexistence of Closely Related Semi‐Arboreal Therates Tiger Beetles (Coleoptera: Cicindelidae)
Source: Ecol Evol. 2025 Nov 17;15(11):e72499. doi: 10.1002/ece3.72499 (PMC12620851; doi:10.1002/ece3.72499)
Supplement: Supplementary file 1 — Data S1: ece372499‐sup‐0001‐Supinfo.zip. [file ECE3-15-e72499-s001.zip › ECE3_72499__author.zip!/ECE3_72499_Acal et al._Query 1_Figure Captions.docx]

**Query 1**

**1. Figure 1-8 file received but caption is missing**

**Figure. 1.** (A) Philippine archipelago. (B) Geographical distribution of sampling sites in southern Philippines (see Table 1 for site details). Circles are sites with species noted in coexistence *T. fulvipennis bidentatus* and *T. fasciatus fasciatus* (blue), *T.* *fulvipennis bidentatus* and *T. fasciatus pseudolatreillei* (green), *T. coracinus coracinus* and *T. fasciatus quadrimaculatus* (yellow), *T. fulvipennis everetti*, and *T. fasciatus quadrimaculatus* (orange). Red triangles represent those sites where only one species was noted (graphics: D. A. Acal).

**Figure.2.** Graphical representation of *Therates* with morphometric parameters (A): total body length (TBL) and right mandible length (RML). Measured leaf parameters (B) (graphics: D. A. Acal).

**Figure. 3.** Phenology (A) and elevational distribution (B) of each studied *Therates* taxa: (A) *T. fulvipennis bidentatus*, (B) *T. fasciatus fasciatus*, (C) *T. coracinus coracinus,* (D) *T. fulvipennis everetti,* (E) *T. fasciatus quadrimaculatus*, and (F) *T. fasciatus pseudolatreillei*. Note:Violin plot widths represent the species occurrences across sites in each month.

**Figure. 4.** Total body length (TBL) and right mandible length (RML) of males and females of the studied coexisting *Therates*: (I) *T. fulvipennis bidentatus* and *T. fasciatus fasciatus*, (II) *T. fulvipennis bidentatus* and *T. fasciatus* *pseudolatreillei*, (III) *T. fasciatus quadrimaculatus* and *T. coracinus coracinus*, (IV) *T. fasciatus quadrimaculatus and T. fulvipennis everetti*. The boxes represent the interquartile range (IQR), with the median shown as the horizontal line within the box: red—females, blue—males. Mann–Whitney U tests: (*p < .001).

**Figure. 5**. Linear regression analysis of right mandible length and total body length across all studied *Therates* species.

**Figure. 6.** Comparison of total body length in *Therates* found in coexisting and non-coexisting populations. *T. fulvipennis everetti* was excluded due to the lack of data from non-coexisting sites. Red—females, blue—males. Mann–Whitney U tests (*p < .001).

**Figure. 7.** Variations on leaf surface area (SA) and leaf aspect ratio (W/L) between leaves chosen by each coexisting *Therates* species.

**Figure. 8.** PCA analysis of *Therates* body size and leaf parameters in coexisting species at each site with variables including average (Avg) and standard deviation (SD) of the total body length (TBL), leaf surface area (SA), and leaf aspect ratio (W/L). For IV coexisting pair, *T. fulvipennis everetti* and *T. fasciatus quadrimaculatus* were collected only in two localities.
